# Serum immunoregulatory proteins as predictors of overall survival of metastatic melanoma patients treated with ipilimumab

**DOI:** 10.1186/2051-1426-3-S2-P96

**Published:** 2015-11-04

**Authors:** Yoshinobu Koguchi, Helena Hoen, Shelly Bambina, Michael Rynning, Richard Fuerstenberg, Zipei Feng, Bernard Fox, Carlo Bifulco, Brendan D Curti, Walter Urba, Christina Milburn, Alan J Korman, Keith S Bahjat

**Affiliations:** 1 Earle A. Chiles Research Institute, Portland, OR, USA; 2 R&D Systems, Minneapolis, MN, USA; 3 Providence Cancer Center, Portland, OR, USA; 4 Providence Portland Medical Center, Portland, OR, USA; 5 Bristol-Myers Squibb, Redwood City, CA, USA; 6 Robert W. Franz Cancer Research Center, Portland, OR, USA

## Background

Treatment with ipilimumab improves overall survival (OS) in patients with metastatic melanoma. Because ipilimumab targets T lymphocytes and not the tumor itself, efficacy may be uniquely sensitive to immunomodulatory factors present at the time of treatment.

## Methods

We analyzed serum from patients with metastatic melanoma (247 of 273, 90.4%) randomly assigned to receive ipilimumab or gp100 peptide vaccine (NCT00094653). We quantified candidate biomarkers at baseline and assessed the association of each with overall survival using univariate and multivariate analyses. Results were confirmed in an independent cohort of similar patients (48 of 52, 92.3%) treated with ipilimumab (NCT00495066).

## Results

Univariate analysis of biomarkers identified chemokine (C-X-C motif) ligand 11 (CXCL11) and soluble MHC class I polypeptide-related chain A (sMICA) as potential predictive biomarkers for ipilimumab but not gp100 therapy for metastatic melanoma. After controlling for baseline covariates, elevated CXCL11 and sMICA were associated with poor OS in ipilimumab-treated patients (log10 CXCL11: hazard ratio (HR), 1.88; 95% CI, 1.14 to 3.12; *P* = 0.014; and log10 sMICA quadratic effect *P* = 0.066; sMICA (≥247 vs < 247): HR, 1.75; 95% CI, 1.02 to 3.01) but not in gp100-treated patients. Multivariate analysis of an independent ipilimumab-treated cohort confirmed the association between log10 CXCL11 and OS (HR, 3.18; 95% CI 1.13 to 8.95; *P* = 0.029), while sMICA was less strongly associated with OS (log10 sMICA quadratic effect *P* = 0.16; sMICA (≥247 vs < 247): HR, 1.48; 95% CI, 0.67 to 3.27).

## Conclusion

Low baseline CXCL11 and sMICA were associated with improved OS in patients with metastatic melanoma after ipilimumab treatment but not vaccine treatment. Thus, pretreatment CXCL11 and sMICA may represent predictors of survival benefit after ipilimumab treatment as well as therapeutic targets. Furthermore, their role in recruiting T regulatory cells (CXCL11) and inhibiting cytolytic effector cells (sMICA) suggests that combination therapies targeting these molecules may synergize with CTLA-4 blockade in patients.

